# Effect of Chinese herbal medicine compound on breast hyperplasia: A protocol of systematic review

**DOI:** 10.1097/MD.0000000000023463

**Published:** 2020-12-04

**Authors:** Hui-lin Guan, Yu Wang, Yi-fang Gui, Chun-lei Zhang

**Affiliations:** a Department of Scientific Research, Mudanjiang Medical University; b Department of Reproduction, Mudangjiang Women and Children Hospital; c Department of Laboratory, The Affiliated Hongqi Hospital of Mudanjiang Medical University, Mudanjiang, China.

**Keywords:** breast hyperplasia, Chinese herbal medicine compound, effect

## Abstract

**Background:**

Data supporting the use of Chinese herbal medicine compound (CHMC) on breast hyperplasia (BH) based on the data from previous studies. However, the results are still contradictory. Thus, this study aims to compare the results obtained for effect on case-controlled study (CCS) of CHMC on BH.

**Methods:**

This study will include CCS assessing the effect of CHMC on BH. A literature search will be carried out in Cochrane Library, MEDLINE, EMBASE, Allied and Complementary Medicine Database, Chinese Biomedical Literature Database, and China National Knowledge Infrastructure from inception to the present. We will not apply language limitation to any electronic database. Study quality will be evaluated using Newcastle-Ottawa Scale, and statistical analysis will be performed using RevMan 5.3 software.

**Results:**

This study will summarize the up-to-date evidence to assess the effect of CHMC on BH.

**Conclusion:**

The results of this study may exert helpful evidence to determine whether CHMC is effective on BH.

**OSF registration number:**

osf.io/3k8ch.

## 1 Introduction

Breast hyperplasia (BH) is a very common benign breast condition, with prevalence increasing each year.^[1–3]^ It is usually detected by chance, and it does not manifest any specific symptoms, except lump or pain.^[4,5]^ It usually develops as the breast changes with age, and often occurs in women over 35 years old.^[6]^ Although it is a benign condition, patients with BH have an increased risk of developing breast cancer.^[7–11]^ Thus, it is very important to manage this issue.

Despite lots of studies have reported that Chinese herbal medicine compound (CHMC) can help treat BH,^[12–26]^ there are still not consistent conclusions among those studies. In addition, no systematic review specifically explores this topic. Therefore, this study will firstly and systematically address the effect of CHMC on BH.

## 2 Methods

### 2.1 Study registration

This study has been registered on OSF (osf.io/3k8ch). It is reported based on the guidelines of Preferred Reporting Items for Systematic Reviews and Meta-Analysis Protocol statement guidelines.

### 2.2 Eligibility criteria

#### 2.2.1 Types of studies

Case-controlled study (CCS) will be selected to evaluate the effect of CHMC on BH.

#### 2.2.2 Types of interventions

Intervention: All participants in the experimental group received CHMC for the treatment of BH.

Comparator: All participants with BH in the control group were given any management, such as no treatment, and placebo.

#### 2.2.3 Types of subjects

Studies assessing rats which were diagnosed with BH will be included in this study.

#### 2.2.4 Types of outcomes

Outcomes include nipple diameter, mammary gland volume, mammary gland mass, mammary lobule number, acinar number in the lobule, serum estradiol, prolactin level, serum superoxide dismutase activity, serum malondialdehyde, and toxicity.

#### 2.2.5 Information sources

A literature search will be conducted at Cochrane Library, MEDLINE, EMBASE, Allied and Complementary Medicine Database, Chinese Biomedical Literature Database, and China National Knowledge Infrastructure since their inception to the present. No language limitation is applied to all electronic databases. In addition, we will also search conference proceedings, dissertations, and reference lists of included studies. A sample of search strategy for Cochrane Library is presented in Table 1. We will also adapt similar search strategy to other electronic databases.

**Table 1:** Search strategy sample of Cochrane Library.

Number	Search terms
1	Mesh descriptor: (postoperative pain) explode all trees
2	((postoperative^∗^) or (pain, postoperative^∗^) or (postoperative pain^∗^) or (persistent postsurgical pain^∗^) or (postoperative^∗^) or (post surgery^∗^) or (pain intensity^∗^)):ti, ab, kw
3	Or 1–2
4	Mesh descriptor: (acupuncture) explode all trees
5	Mesh descriptor: (electroacupuncture) explode all trees
6	((needling^∗^) or (acupionts^∗^) or (fire needle^∗^) or (auricular needle^∗^) or (auriculo-acupuncture^∗^) or (ear acupuncture^∗^) or (scalp acupuncture^∗^) or (dermal needle^∗^) or (abdominal acupuncture^∗^)):ti, ab, kw
7	Or 4–6
8	MeSH descriptor: (randomized controlled trials) explode all trees
9	((RCT^∗^) or (randomly ^∗^) or (random^∗^) or (blind ^∗^) or (allocation ^∗^) or ((control^∗^) or (placebo ^∗^) or (sham^∗^) or (clinical study^∗^) or (clinical trials^∗^) or (controlled study^∗^) or (controlled trial^∗^)):ti, ab, kw
10	Or 8–9
11	3 and 7 and 10

lateral[title] AND epicondylitis[title] 115 arthroscop^∗^[title] AND lateral[title] AND epicondylitis[title] lateral[title] AND epicondylitis[title] AND surgery[title] OR surgical[title] AND management[title] percutaneous[title] AND lateral[title] AND epicondylitis[title] tennis[title] AND elbow[title] AND surgery[title] common[title] AND extensor[title] AND release[title].

### 2.3 Selection of studies

Before the selection of studies, we will build standard eligibility criteria to identify all searched studies. Two authors will independently scan the titles and abstracts. All irrelevant studies will be excluded, and we will read full texts of remaining studies. We will present process of study selection in a flowchart. Two authors will perform all study selection, respectively. Any different opinions will be solved by consensus with a third author.

### 2.4 Data extraction and management

Two authors will independently extract data based on the predefined data collection form. It comprises of title, first author, time of publication, study setting, subject characteristics (sample size, age, sex), study methods, intervention and control details (intervention types, dosage, frequency, and duration), outcomes, and funding information. Any disagreements about data extraction will be resolved by discussion with another author.

### 2.5 Study quality assessment

Two authors will independently evaluate the risk of bias of the retrieved studies using Newcastle-Ottawa Scale. A third author will help to solve any divergences about study quality assessment between 2 authors.

### 2.6 Statistical analysis

We will perform statistical analysis using RevMan 5.3 software (Cochrane Community, London, UK.). In this study, we will exert continuous data as mean difference and 95% confidence intervals (CIs); and dichotomous data as risk ratio and 95% CIs. We will identify heterogeneity among included studies using *I*^2^ statistic. *I*^2^ ≤ 50% indicates acceptable heterogeneity, whereas *I*^2^ > 50% means indication of obvious heterogeneity, respectively. If acceptable heterogeneity is identified, we will use a fixed-effect model. On the other hand, if substantial heterogeneity is detected, we will utilize a random-effect model. Meanwhile, we will also conduct subgroup analysis to explore the reasons which are responsible for the significant heterogeneity. We will not pool the outcome data and will not carry out meta-analysis if significant heterogeneity still exists after subgroup analysis. Instead, we will report narrative summary description in accordance with the Guidance on the Conduct of Narrative Synthesis in Systematic Reviews.

### 2.7 Additional analysis

#### 2.7.1 Subgroup analysis

If sufficient studies are included, we will carry out subgroup analysis according to the different study information, treatments, controls, and outcomes.

#### 2.7.2 Sensitivity analysis

When necessary, sensitivity analysis will be performed to identify stability of pooled outcome results by excluding low quality studies.

#### 2.7.3 Reporting bias

If >10 CCS are entered in this study, we will conduct funnel plot and Egger regression test to explore any possible reporting bias.^[27,28]^

#### 2.7.4 Ethics and dissemination

This study will not need ethical approval as no primary data will be collected. This study will be disseminated via a peer-reviewed journal or conference meetings.

## 3 Discussion

In this study, we firstly investigate the effect of CHMC on BH. Only the evidence from eligible CCS will be considered for inclusion. Methodological quality of all included studies will be appraised by 2 authors, respectively. The interpretation of study methodological quality should take their designs, methods, and data analyst into account. The contribution of this study will supply evidence on both external validity of these results, as well as its usage to the clinical practice.

## Author contributions

**Conceptualization:** Hui-lin Guan, Yi-fang Gui, Chun-lei Zhang.

**Data curation:** Hui-lin Guan, Yu Wang, Yi-fang Gui, Chun-lei Zhang.

**Formal analysis:** Hui-lin Guan, Yu Wang, Yi-fang Gui, Chun-lei Zhang.

**Investigation:** Yi-fang Gui.

**Methodology:** Hui-lin Guan, Yu Wang, Chun-lei Zhang.

**Project administration:** Yi-fang Gui.

**Resources:** Hui-lin Guan, Yu Wang, Chun-lei Zhang.

**Software:** Hui-lin Guan, Yu Wang, Chun-lei Zhang.

**Supervision:** Yi-fang Gui.

**Validation:** Hui-lin Guan, Yu Wang, Yi-fang Gui, Chun-lei Zhang.

**Visualization:** Hui-lin Guan, Yi-fang Gui, Chun-lei Zhang.

**Writing – original draft:** Hui-lin Guan, Yu Wang, Yi-fang Gui, Chun-lei Zhang.

**Writing – review & editing:** Hui-lin Guan, Yu Wang, Chun-lei Zhang.
